# Liquid Membranes as a Tool for Chemical Speciation of Metals in Natural Waters: Organic and Inorganic Complexes of Nickel

**DOI:** 10.3390/membranes8020019

**Published:** 2018-04-15

**Authors:** Cristina Vergel, Carolina Mendiguchía, Carlos Moreno

**Affiliations:** Department of Analytical Chemistry, Faculty of Marine and Environmental Sciences, University of Cádiz, 11510 Puerto Real (Cádiz), Spain; cristina.vergel@uca.es (C.V.); carolina.mendiguchia@uca.es (C.M.)

**Keywords:** natural water, trace metal, nickel, pre-concentration, speciation, liquid membranes

## Abstract

The different species of nickel present in natural waters exhibit different transport behaviour through bulk liquid membranes (BLMs). This fact has been used to design and optimise a separation/pre-concentration system applicable to separate labile and non-labile nickel fractions. A hydrazone derivative—1,2-cyclohexanedione bis-benzoyl-hydrazone (1,2-CHBBH) dissolved in toluene/dimethyl formamide (2% DMF)—was used as a chemical carrier of nickel species, from an aqueous source solution (sample) to a receiving acidic solution. Both chemical and hydrodynamic conditions controlling the transport system were studied and optimised. Under optimum conditions, variations in the transport of nickel ions as a function of organic (humic acids) and inorganic (chloride ions) ligands were studied. Relationships between the permeability coefficient (*P*) or recovery efficiency (%R) and the concentrations of ligands and nickel species were analysed using Winhumic V software. A negative correlation between P and the concentration of organic nickel complexes was found, suggesting that only labile nickel species are transported through the liquid membrane, with non-labile complexes remaining in the water sample; allowing for their separation and subsequent quantification in natural waters.

## 1. Introduction

Chemical speciation of trace metals in natural waters is currently a hot topic in environmental chemistry. Its importance is due to trace metals are no biodegradable remaining in the environment for long periods distributed between the different environmental compartments [1]. Once they reach the aquatic media, dissolved trace metals mainly appear as free hydrated ions or complexed with organic or inorganic ligands. The distribution of these chemical forms depends mainly on the total metal concentration, pH and redox potential, as well as the concentration of ligands. Because of the different properties and biological availability of each species, this distribution may be associated with different toxicological effects on aquatic organisms [2]. In fact, determination of total metal concentrations very often does not provide the information required to assess metal bioaccumulation and toxicity; thus, it is necessary to analyse the different metal species present in aquatic systems. A very useful and simple alternative takes advantage of the distinction between labile and non-labile chemical forms, which implies an operational definition of the species and therefore separation is dependent on the analytical method used, the so-called “analytical windows.” Nevertheless, in dynamic metal speciation analysis with non-equilibrium techniques, in addition to kinetic windows and thermodynamic considerations, the reactions produced in the bulk solution and in the interfaces (surface of an electrode, organism or a selective membrane) should be taken into account. Thus, in this work the IUPAC (International Union of Pure and Applied Chemistry) definition of the term “labile” for interfacial processes is used, as species which its association/dissociation kinetics is so fast that its flux through the interface is controlled by the coupled diffusion of metal (M) and complex (ML) [3]. Most of the works conclude that labile metal species consist of free hydrated ions or easily exchangeable complexes, mainly formed with inorganic ligands such as chloride and sulphate and so forth, while non-labile species are usually organic complexes, more difficult to dissociate [4,5]. The typical range of dissolved organic carbon (about one half of dissolved organic matter) in natural waters may vary from 0.2 to 60 mg·L^−1^ and even clear water contains at least a small fraction of organic matter, typically in the range 1–3 mg·L^−1^ [6,7].

Most of these organic complexes are formed by humic substances, such as humic (HA) and fulvic acids (FA), which display an affinity for metal ions that generally corresponds to the Irving-William series: Cu^2+^ > Ni^2+^ > Zn^2+^ > Co^2+^ > Cd^2+^ > Ca^2+^ > Mg^2+^ [8]. Nickel speciation in natural waters is strongly dependent on the concentration and nature of the dissolved organic matter [7]; and, typically, approximately 50–70% of dissolved Ni has been reported to form organic complexes in different natural waters [9,10,11].

Several techniques have been proposed to quantify the concentration of the different species of trace metals present in natural waters: mostly involving the use of an electroanalytical device or an advanced separation method, followed by an additional analytical technique [12,13,14,15]. Nevertheless, recent studies suggest that competitive ligand–stripping voltammetry methods overestimate metal complexation by DOM (Dissolved organic Matter) and therefore underestimate metal concentration in comparison with model predictions (WHAM VII) [16]. Among others separation methods, solid phase and liquid-liquid extractions have been extensively used, while recently, liquid membranes have been described as a clean, useful and powerful alternative to these classical methods [17,18].

The applicability of liquid membranes to the analysis of fresh and sea water has been demonstrated for different metals, including nickel [17,18,19,20,21,22,23,24]; however, only a few examples of its application as a fractionation or speciation tool are present in the literature and these deal mostly with copper [17,25,26,27,28,29,30,31].

In the present work, a bulk liquid membrane system has been developed to separate different fractions of nickel (labile and non-labile) in natural water as a simple alternative to electrochemical methodologies. As a chemical carrier, we used a hydrazone derivative, 1,2-cyclohexanedione bis-benzoyl-hydrazone (1,2-CHBBH) in toluene, which has a demonstrated capacity for nickel separation by solvent extraction [32].

## 2. Material and Methods

### 2.1. Reagents and Solutions

All chemicals used were reagent grade unless otherwise stated. Aqueous nickel solutions were prepared from a commercial standard solution of 1000 mg·L^−1^ from Merck (Darmstadt, Germany). Nitric acid (65%), sodium chloride, sulfuric acid (95–97%), sodium hydroxide and toluene were obtained from Scharlab (Barcelona, Spain). Buffer solutions were prepared using *N*-(2-hydroxyethyl)piperazine-*N*′-(2-ethanesulfonic acid) (HEPES) from Biochemical (Barcelona, Spain). *N*,*N*-Dimethylformamide (DMF), ethanol (96%) and perchloric acid (60%) were obtained from Merck (Darmstadt, Germany). Hydrochloric acid was purchased from JT Baker (Phillipsburg, NJ, USA). Humic acid sodium salt was obtained from Aldrich (Steinheim, Germany). The active component of the liquid membrane, 1,2-cyclohexanedione bis-benzoylhydrazone (1,2-CHBBH), was synthesised as previously described [33]. All solutions were prepared using deionised water (18 MΩ·cm^−1^) from a Milli-Q analytical reagent grade water purification system (Millipore, Bedford, MA, USA).

Solutions used as a source phase in optimization experiments consisted of 1 mg·L^−1^ Ni(II) and 35 g·L^−1^ NaCl. These solutions were adjusted to pH 8 using HEPES and sodium hydroxide. Appropriate amounts of sodium chloride and humic acid sodium salt were added to source solutions, to investigate the effects of organic and inorganic ligands.

### 2.2. Procedures

To maximise the transport of nickel species through the membrane, we studied the influence of the concentration of organic reagent in the membrane, acid concentration in the receiving solution and the stirring rate on species transport. In order to assess its applicability to separate different nickel fractions in natural waters, the behaviour of the system was evaluated in the presence of different concentrations of the most representative ligands, for example, Cl^−^ (inorganic ligand) and HA (organic ligand) in the source phase. Both synthetic and real seawater samples were used. Each transport experiment was done, at least, in triplicate and the results obtained were compared with the theoretical concentrations of free and bound nickel in the sample, calculated using the software program WINHUMIC V including the original WHAM Ssed10.dbs database (http://www.lwr.kth.se/english/OurSoftWare/WinHumicV/index.htm) [34].

### 2.3. Bulk Liquid Membrane System

Liquid membrane experiments were carried out using a homemade Teflon beaker-in-a-beaker type cell, as described elsewhere [35]. The system consisted of two concentric beakers containing 53 mL of sample solution (external beaker) and 11.5 mL of acidic receiving solution (internal beaker). Both aqueous solutions were in contact through a liquid membrane, consisting of an organic solution of 1,2-CHBBH dissolved in toluene (2% DMF), which allows selective chemical pumping of nickel species from the sample to the receiving solution. The volume of organic solution was 7 mL and was chosen to be as small as possible in order to maximise the transport rate. Solutions were stirred with a magnetic stirrer (Agimatic SD model, P Selecta, Barcelona, Spain).

The general reaction for the extraction of free nickel ions with 1,2-CHBBH can be expressed as follows [32,33,36,37]:

Ni^2+^_(ac)_ + 2 HR _(org)_ ↔ NiR_2 (org)_ + 2 H^+^_(ac)_

where HR represents the acidic extractant reagent 1,2-CHBBH.

The transport efficiency was quantified by measuring the variation of nickel concentration in the receiving solution and in terms of mass flux across the membrane, measured as the permeability coefficient (*P*), according to the equation [38]:

$$-\ln\left[Ni^{2+}\right]=\frac{S}{V_{s}}Pt-\ln{\left[Ni^{2+}\right]}_{0}$$

where *S* is the effective membrane area, *V_s_* the volume of the source solution and [Ni^2+^]_0_ and [Ni^2+^] are total nickel concentrations in the source solution at time 0 and *t*, respectively.

Nickel concentrations in the receiving solutions were measured using an Atomic Absorption Spectrometer (AAS), model Solaar M Series (Thermo, Waltham, MA, USA), while the pH was measured using a model 2001 pH-meter with a combined glass Ag/AgCl electrode (Crison, Barcelona, Spain). By using AAS, the potential interferences caused by other transported metals can be overcame.

### 2.4. Effect of Ligands on the BLM System

HA (15–100 mg·L^−1^) and NaCl (5–25 g·L^−1^ Cl^−^) were added to different source solutions containing 1 mg·L^−1^ nickel, in order to evaluate the effects of both organic and inorganic ligands on the transport of nickel through the membrane. Both, the permeability coefficient and recovery efficiency (measured as the percentage of nickel recovered in the receiving solution) were estimated as a function of ligand concentrations. To confirm the results obtained with organic matter, experiments with humic acids were repeated but, before membrane experiments, organic matter was removed by oxidation with UV radiation for 90 min using an A705 UV digester (Metrohm, Herisau, Switzerland), after the addition of 100 µL H_2_O_2_ to each sample. Permeability coefficients were calculated after UV digestion and compared with values obtained in the presence of organic matter.

Analysis of a spiked (1 mg·L^−1^ Ni^2+^) real seawater sample from the Gulf of Cádiz after a UV digestion was performed to evaluate the influence of sample matrix on the recovery efficiency.

Organic matter was measured as dissolved organic carbon using a multi N/C 3100 analyser (Analytik Jena, Jena, Germany).

## 3. Results and Discussion

### 3.1. Optimization of the Bulk Liquid Membrane System

**Figure 1:**
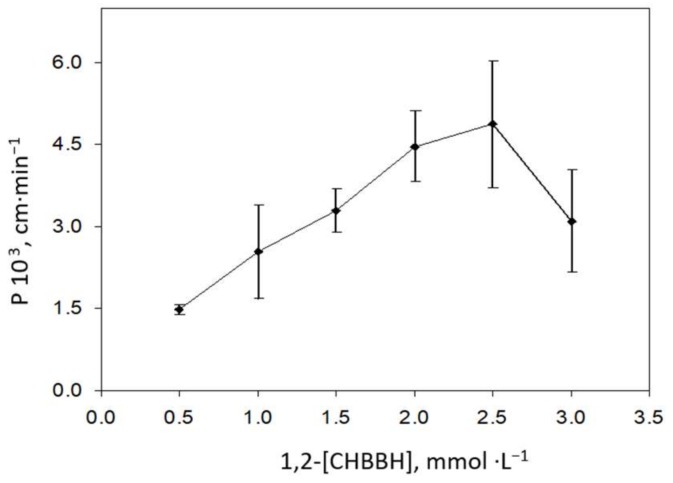
Variation of the permeability coefficient with the 1,2-CHBBH concentration in the organic phase. Source solution: 1 mg·L^−1^ Ni^2+^, 35 g·L^−1^ NaCl, 0.25 mol·L^−1^ HEPES, pH 8.

The dependence of the permeability coefficient of nickel on 1,2-CHBBH concentration (between 0.5 and 3 mmol·L^−1^) is shown in Figure 1. As observed, a maximum *P* value was reached for 2.5 mmol·L^−1^ 1,2-CHBBH and then lower *P* value was observed, together with microprecipiation in the organic phase. Therefore, this concentration of 1,2-CHBBH was selected for further experiments.

**Figure 2:**
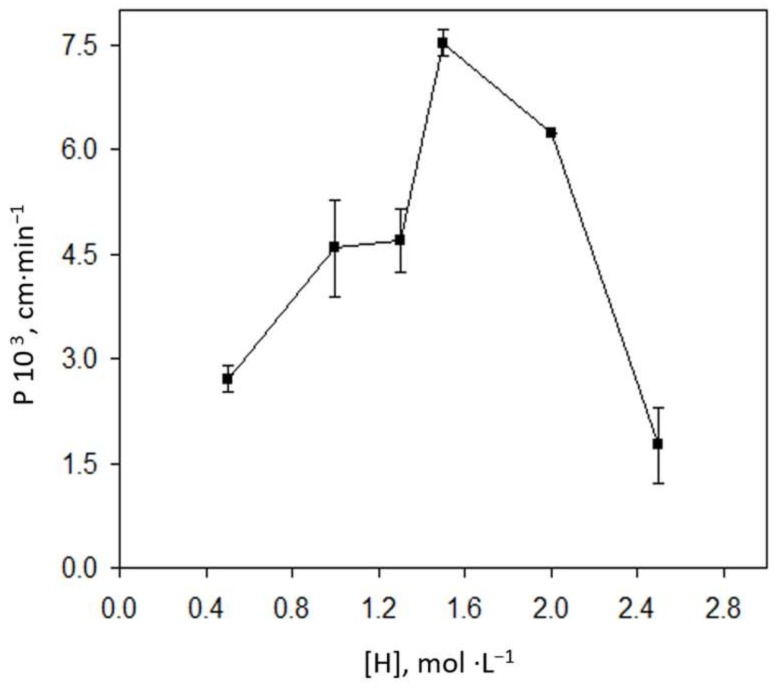
Effect of the hydrochloric acid concentration in the receiving solution on the permeability coefficient. Source solution: 1 mg·L^−1^ Ni^2+^, 35 g·L^−1^ NaCl, 0.25 mol·L^−1^ HEPES, pH 8.

The acid concentration in the receiving solution was also varied between 0.5 and 2.5 mol·L^−1^, indicating a gradual increase in *P* values at acidities up to 1.5 mol·L^−1^ (Figure 2), due to the acidic character of the extraction mechanism. Higher acidity caused decreased nickel transport through the membrane, probably due to carrier instability. The study was carried out with various strong acids such as nitric, hydrochloric and perchloric acid; and higher permeability coefficients were obtained with HCl and HNO_3_, although a reddish precipitate appeared in the organic membrane with HNO_3_, probably due to the formation of a ternary organic species with NO_3_^−^. Consequently, 1.5 mol·L^−1^ HCl was selected as the optimum receiving solution.

**Figure 3:**
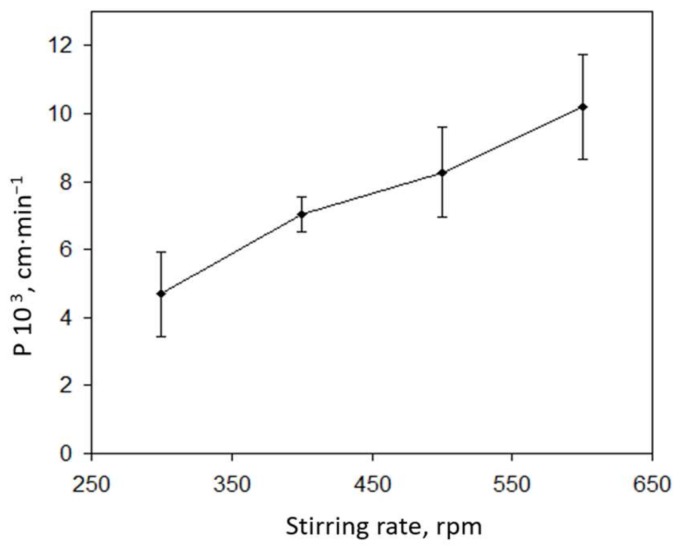
Variation of the permeability coefficient with the stirring rate. Source solution: 1 mg·L^−1^ Ni^2+^, 35 g·L^−1^ NaCl, 0.25 mol·L^−1^ HEPES, pH 8.

Once chemical variables were optimised, the effects of hydrodynamic conditions were investigated by stirring the three liquid phases simultaneously. As can be seen in Figure 3, permeability coefficients were observed to increase with increasing stirring rates up to 600 rpms: at higher rates, the liquid membrane was broken, resulting in mixing of both aqueous solutions. Therefore, to ensure membrane integrity while maximizing permeability, a stirring rate of 500 rpms was selected as optimal.

**Figure 4:**
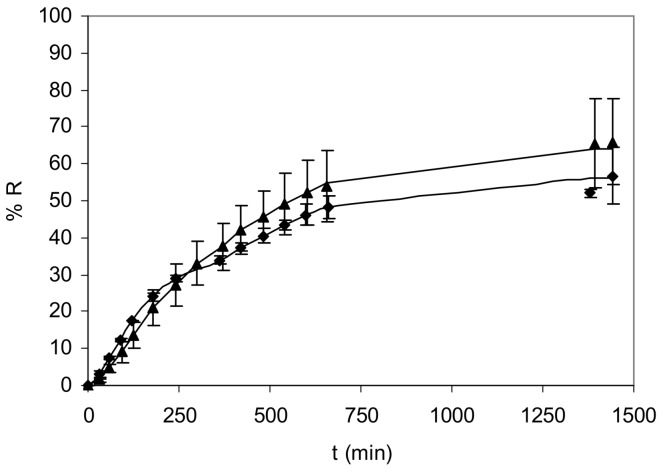
Temporal variation of recovery efficiency for a synthetic (35 g·L^−1^ NaCl) (◆) and a real seawater sample (▲).

Using these optimum conditions, the recovery efficiency, measured as the percentage of nickel recovered in the receiving solution, was evaluated for 24 h experiments. As Figure 4 shows, recovery efficiency increases up to 50.5 ± 3.2% after 11 hours of experiment when transport is almost finished.

### 3.2. Effects of Ligands on Nickel Transport

**Figure 5:**
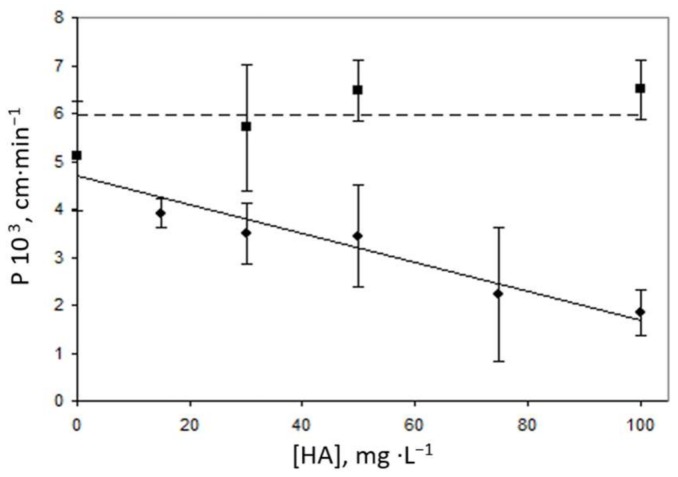
Effect of HA concentration on the permeability coefficient. (●) sample without treatment; (■) sample after UV treatment for elimination of organic matter (dashed line indicates average value). Source solution: 1 mg·L^−1^ Ni^2+^, 35 g·L^−1^ NaCl, 0.25 mol·L^−1^ HEPES, pH 8. Receiving solution: 1.5 mol·L^−1^ HCl. Organic phase: 2.5 mmol·L^−1^ 1,2-CHBBH in toluene (2% DMF). Stirring rate: 500 rpm.

When permeability coefficient was measured in the absence of Cl^−^, a value of 7.63 (±0.43) 10^−3^ cm·min^−1^ was obtained. If chloride ions were present in the samples, a decrease in *P* was observed due to the formation of monovalent NiCl^+^ complexes, which could exhibit a lower transport rate or even not be extracted by 1,2-CHBBH. When Cl^−^ concentrations varied between 5 and 25 g·L^−1^, *P* value was nearly independent of Cl^−^ concentration and a mean permeability coefficient of 5.64 (±0.36) 10^−3^ cm·min^−1^ was obtained. For seawater conditions, the observed decrease in permeability coefficient was 26%, consistent with the concentration of NiCl^+^ in the samples (see Table 1). As Figure 5 shows, differences in the recovery efficiency were not observed when a real seawater sample was analysed after a UV digestion to destroy organic complexes.

As shown in Figure 5, a decrease in permeability coefficient was observed when, in addition to Cl^−^, HA was added to the source solution, probably due to the formation of organic complexes which cannot permeate across the liquid membrane. In fact, at 100 mg·L^−1^ HA, *P* values were decreased by greater than 60%. The dependence of this decrease in *P* on HA concentration was confirmed by analysing samples containing the same concentrations of HA but previously digested with UV radiation to eliminate the organic matter. In this case, while pH values kept almost invariable, values of *P* obtained were similar to those obtained in the absence of HA, with an average value of 5.95 (±0.68) 10^−3^ cm·min^−1^ for 15–100 mg·L^−1^ HA.

Table 1 shows the theoretical distribution of nickel species in an aquatic system under the studied conditions, calculated using the WINHUMIC V software (KTH Royal Institute of Technology, Stockholm, Sweden). As observed, the presence of Cl^−^ and HA mostly resulted in the formation of NiCl^+^ and anionic Ni-HA complexes which could be not transported through the liquid membrane. Permeability coefficients significantly correlated (α = 0.05) with the concentration of organic complexes, with a Spearman coefficient of 0.943.

In addition to P, the recovery efficiency of transport was also calculated for different concentrations of humic acids. Although the time required to achieve maximum transport was not affected by the presence of humic acids, a decrease in the efficiency was observed, as can be seen in Table 2, which includes data from 11 h of transport. This fact may be explained by the formation of anionic non-labile organic complexes, which cannot be transported through the liquid membrane.

As a consequence of these results, we concluded that only the most labile nickel species are transported through the membrane, while stable complexes, mainly organic complexes, remain in the water sample, enabling separation of the different fractions.

A significant negative correlation (α = 0.05) was observed between the recovery efficiency and the concentration of organic matter, measured both as dissolved organic carbon (DOC) and Ni-HA complexes, with a Spearman coefficient of −0.900. Based on these results, a linear relationship was established to estimate the recovery efficiency (%R) as a function of DOC:

(%R) = −0.6 [DOC] + 50.5

### 3.3. Measuring the Concentration of Nickel Species in Real Samples

**Figure 6:**
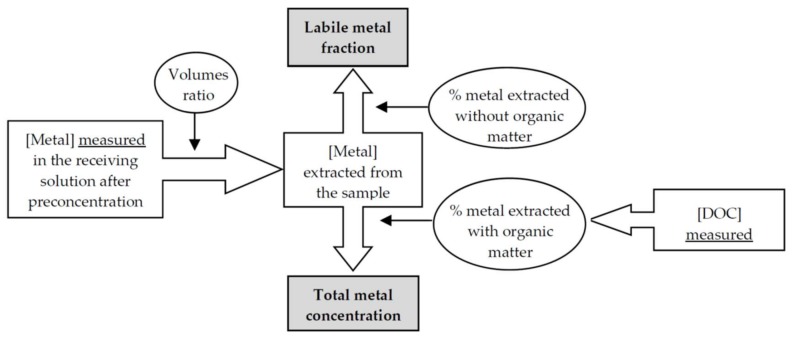
Schematic procedure for nickel speciation in water samples.

Figure 6 shows in outline, as an example, the liquid membrane-based procedure proposed to measure the different nickel fractions present in a water sample. After pre-concentration, the nickel concentration is measured in the receiving solution and, using volumes ratio, the nickel concentration extracted from the sample is calculated. Then, by using the %R in the absence of organic matter (which is always 50.5%) an estimation of labile metal fraction is obtained. In addition, after measuring DOC concentration, we may estimate the %R for a sample using the linear relationship shown above. Then, the total metal concentration is calculated using this %R.

Finally, the difference between labile and total nickel concentrations can be used to estimate the non-labile nickel fraction.

## 4. Conclusions

We evaluated a novel bulk liquid membrane system to separate nickel fractions from natural waters. Optimum conditions to maximise nickel transport through the membrane were determined to be: 2.5 mmol·L^−1^ 1,2-CHBBH in the organic phase; 1.5 mol·L^−1^ HCl in the receiving solution; and a stirring rate of 500 rpms. Under these conditions, the permeability coefficient and recovery efficiency were dependent on the humic acid concentration, resulting in a decrease in nickel transport as ligand concentration increased. This decrease was related to the formation of non-labile complexes between nickel and humic acids, which cannot be transported through the membrane, as evidenced by the correlation between the permeability coefficient and theoretical NiHA concentrations obtained from Winhumic V software. Our results suggest that the liquid membrane system can be applied to separate labile and non-labile nickel fractions in natural water samples, even seawater. Speciation could be performed after only one extraction of the nickel present in the samples and previous analyses of the dissolved organic carbon concentration.

## Figures and Tables

**Table 1:** Nickel species' distribution in the source solution for different HA concentrations was calculated with the WinHumicV software. Source solution: 1 mg·L^−1^ Ni^2+^, 35 g·L^−1^ NaCl, 0.25 mol·L^−1^ HEPES, pH 8.

Humic Acid, mg·L^−1^	NiHA		Ni(OH)_2_		Ni^2+^		NiCl^+^		NiOH^+^	
	mol·L^−1^	%	mol·L^−1^	%	mol·L^−1^	%	mol·L^−1^	%	mol·L^−1^	%
0	0	0	2.9 × 10^−9^	0	1.3 × 10^−5^	74	4.3 × 10^−6^	26	6.8 × 10^−8^	0
15	8.1 × 10^−7^	5	2.7 × 10^−9^	0	1.2 × 10^−5^	70	4.0 × 10^−6^	24	6.3 × 10^−8^	0
30	1.5 × 10^−6^	9	2.5 × 10^−9^	0	1.1 × 10^−5^	67	3.8 × 10^−6^	23	5.9 × 10^−8^	0
50	2.4 × 10^−6^	15	2.3 × 10^−9^	0	9.9 × 10^−6^	63	3.4 × 10^−6^	22	5.4 × 10^−8^	0
75	3.3 × 10^−6^	21	2.1 × 10^−9^	0	9.0 × 10^−6^	58	3.1 × 10^−6^	20	4.8 × 10^−8^	0
100	4.0 × 10^−6^	27	1.7 × 10^−9^	0	8.1 × 10^−6^	54	2.8 × 10^−6^	19	4.4 × 10^−8^	0

**Table 2:** Recovery efficiency values for different HA concentrations. Source solution: 1 mg·L^−1^ Ni^2+^, 35 g·L^−1^ NaCl, 0.25 mol·L^−1^ HEPES, pH 8.

Humic Acid, mg·L^−1^	DOC, mg·L^−1^	Recovery Efficiency, %	Standard Deviation
0	0	50.5	3.2
15	3.65	48.0	2.6
50	12.20	47.2	4.4
75	18.58	33.7	7.5
100	25.95	37.5	6.8
